# The Integrated Analysis Identifies Three Critical Genes as Novel Diagnostic Biomarkers Involved in Immune Infiltration in Atherosclerosis

**DOI:** 10.3389/fimmu.2022.905921

**Published:** 2022-05-18

**Authors:** Zhen Ye, Xiao-kang Wang, Yun-hui Lv, Xin Wang, Yong-chun Cui

**Affiliations:** ^1^Center for Cardiovascular Experimental Study and Evaluation, Fuwai Hospital, Chinese Academy of Medical Sciences and Peking Union Medical College, State Key Laboratory of Cardiovascular Disease, National Center for Cardiovascular Diseases, Beijing Key Laboratory of Pre-clinical Research and Evaluation for Cardiovascular Implant Materials, Beijing, China; ^2^Department of Pharmacy, Suqian First Hospital, Suqian, China

**Keywords:** atherosclerosis, immune infiltration, diagnosis, biomarker, GEO datasets, machine learning

## Abstract

Atherosclerosis (AS), a chronic inflammatory disease of the blood vessels, is the primary cause of cardiovascular disease, the leading cause of death worldwide. This study aimed to identify possible diagnostic markers for AS and determine their correlation with the infiltration of immune cells in AS. In total, 10 serum samples from AS patients and 10 samples from healthy subjects were collected. The original gene expression profiles of GSE43292 and GSE57691 were downloaded from the Gene Expression Omnibus database. Least absolute shrinkage and selection operator regression model and support vector machine recursive feature elimination analyses were carried out to identify candidate markers. The diagnostic values of the identified biomarkers were determined using receiver operating characteristic assays. The compositional patterns of the 22 types of immune cell fraction in AS were estimated using CIBERSORT. RT-PCR was performed to further determine the expression of the critical genes. This study identified 17 differentially expressed genes (DEGs) in AS samples. The identified DEGs were mainly involved in non-small cell lung carcinoma, pulmonary fibrosis, polycystic ovary syndrome, glucose intolerance, and T-cell leukemia. FHL5, IBSP, and SCRG1 have been identified as the diagnostic genes in AS. The expression of SCRG1 and FHL5 was distinctly downregulated in AS samples, and the expression of IBSP was distinctly upregulated in AS samples, which was further confirmed using our cohort by RT-PCR. Moreover, immune assays revealed that FHL5, IBSP, and SCRG1 were associated with several immune cells, such as CD8 T cells, naïve B cells, macrophage M0, activated memory CD4 T cells, and activated NK cells. Overall, future investigations into the occurrence and molecular mechanisms of AS may benefit from using the genes FHL5, IBSP, and SCRG1 as diagnostic markers for the condition.

## Introduction

Atherosclerosis (AS) is a cardiovascular disease caused by the thickening and hardening of arterial walls due to the accumulation of cells, cholesterol, and extracellular matrix (1, 2). Epidemiological research has indicated that hypertension, smoking, diabetes mellitus, and hypercholesterolemia are the main dangerous elements for AS and associated illness processes (3, 4). Besides this, according to laboratory and clinical data results, the combined impacts of aging and inflammation result in a high occurrence rate of AS (5). Though a great improvement in its treatment has been made, the danger of co-morbidities in AS is still serious (6, 7). AS is a chronic and complicated course involving many cells and molecular alterations. Furthermore, it is estimated that approximately 50% of the risk for atherosclerosis is genetically determined (8, 9). Therefore, the diagnosis of AS requires more novel biomarkers and forecasting models to be authenticated.

Data mining has been applied in many fields, containing sequence analysis, microarray gene expression, single-nucleotide polymorphism inspection as well as the analysis of genomic loss and amplification (copy number variation) (10, 11). With the help of microarrays, integration bioinformatics makes scholars confirm distinctively expressed targeted genes soon between AS specimens in one test (12, 13). AS diagnostic markers could be derived from aberrantly expressed genes. TP53, MAPK1, STAT3, HMOX1, and PTGS2 have been identified to be the underlying diagnostic biomarkers for AS according to the identification of a gene expression profile analysis (14, 15). Preclinical AS may be diagnosed using intercellular adhesion molecule-1 (16, 17). At the same time, diagnostic values will be increased by a large margin if many biomarkers are combined in one model.

In this study, the GSE43292 and GSE57691 datasets published by Bricca et al. and Erik et al. were reanalyzed (18, 19). We screened the abnormally expressed genes in AS samples from big data analysis. Machine learning algorithms were applied to screen and confirm the diagnostic biomarkers for AS. In this paper, CIBERSORT was first applied to quantify the immune cells of specimens of AS and normal tissues based on the gene expression profile. We also looked into the interaction between the detected biomarkers and the infiltrating immune cells to provide the groundwork for future studies.

## Materials and Methods

### Microarray Datasets

The study downloaded the original gene expression profiles GSE43292 and GSE57691 from the Gene Expression Omnibus (GEO) database (https://www.ncbi.nlm.nih.gov/geo/). In total, 32 non-AS and 32 AS arterial samples were included in the GSE43292 dataset. The GSE57691 dataset included 10 non-AS and 9 AS arterial specimens.

### Patients and Samples

A total of 20 patients from the Suqian First Hospital, with admission dates from 2020 to 2022 were registered and separated into the AS group (*n* = 10) and the control group (*n* = 10). Blood specimens from the two groups were gathered through venipuncture, and the serum was obtained through centrifugation at 1,500*g* under 4°C and then reserved under −80°C for further use. The study protocol was authorized by the clinical study ethics committee of the Suqian First Hospital. Every method used was according to the related guidelines and regulations. Every participant has signed an informed consent form.

### Data Processing and Screening of Differentially Expressed Genes

Using the combat function of the SVA package, GSE43292 and GSE57691 were combined into a metadata cohort, and batch effects were removed. Comparing the AS arterial samples with the non-AS arterial samples, the “limma” package was applied for the identification of differentially expressed genes (DEGs) with threshold of |fold change (FC)| >1.2 and *P*-value <0.01.

### Functional Enrichment Analysis

The “ClusterProfiler” package of R software was applied in function gathering analysis, and there were significant (*q*-values less than 0.01) biological processes and Kyoto Encyclopedia of Genes and Genomes (KEGG) pathways used in this study (20). Disease Ontology (DO) gathering analysis was operated on DEGs with the “clusterProfiler” and DOSE packages of R.

### Candidate Diagnostic Biomarkers

Support vector machine recursive feature elimination (SVM-RFE) and least absolute shrinkage and selection operator (LASSO) were employed to classify the diagnostic indicators of AS. The “Glmnet” package was employed to analyze the binomial response type and 1 alpha value. In addition, as a supervised machine learning means to support vectors, the SVM explores the optimal variables by clearing the character vectors produced by the SVM. The SVM sorter of R package e1071 was used in the classification analysis of the selective markers for AS diagnosis; *k* = 5 was set for *k*-fold cross-validation. The halving parameter mentioned above was set as 100.

### Evaluation of Immune Cell Infiltration

Gene expression matrix information was uploaded to CIBERSORT (https://cibersort.stanford.edu/) to assess the enrichment of immune invasions, and we got the immune cell infiltrate matrix. Next, the “corrplot” package was used to make a correlation heat map to see the pertinence of 22 kinds of infiltrated immune cells.

### Association Between Critical Genes and Infiltrating Immune Cells

The relationship of the confirmed genes to the standards of infiltrated immune cells was found with Spearman’s rank analysis using R software. The consequent relationships were seen by chart technology using the “ggplot2” package (21).

### Quantitative Real-Time PCR Assay

Total RNA was separated with TRIZOL reagent based on the protocol from the manufacturer (Invitrogen). The RNA was reverse-transcribed with SuperScript First Strand cDNA System (Invitrogen) based on the instructions from the manufacturer. SYBR Green RT-PCR Master Mix and 1.0 μl of cDNA were used in the PCR amplification using Applied Biosystems 7900HT (Applied Biosystems, Takara). The results were gathered and studied by SDS2.3 Software (Applied Biosystems). The expression standard of every candidate gene was the internal standard compared with that of GAPDH. This relative quantitative data was conveyed using the 2^-ΔΔCt^ method. Every test was repeated 3 times. The primers are shown in **Supplementary Table S1**.

### Statistical Analysis

Statistical analyses were performed with R software v3.5.0 (R Core Team, MA, USA) and GraphPad Prism v7.00 (GraphPad Software Inc., La Jolla, CA, USA). R’ “glmnet” package was used to finish the LASSO regression analysis, while the e1071 package ran the SVM algorithm. The significance of distinction in the two groups was calculated with Student’s *t*-test. All *P <*0.05 were regarded as statistically evident.

## Results

### Identification of the Dysregulated Genes in AS Patients

**Figure 1:**
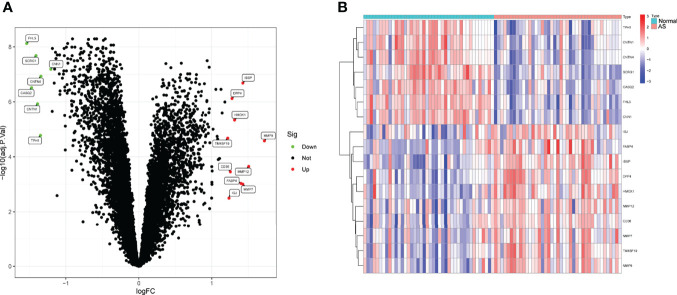
Differentially expressed genes (DEGs) between AS samples and control samples. **(A)** Volcanic map. Colored dots indicate genes that have been up- or downregulated, respectively. **(B)** Heat map of the DEGs.

To screen the dysregulated genes in AS, we analyzed the GSE43292 and GSE57691 datasets using the “limma” package in R software. Through a volcano plot and a hierarchical cluster, we identified a total of 17 DEGs (fold change >1.2), among which 7 (FHL5, CASQ2, SCRG1, CNTN1, TPH1, CNTN4, and CNN1) were downregulated and 10 (TM4SF19, IGJ, CD36, DPP4, HMOX1, FABP4, IBSP, MMP7, MMP9, and MMP12) were upregulated (**Figures 1A, B****)**.

### Functional Correlation Analysis

**Figure 2:**
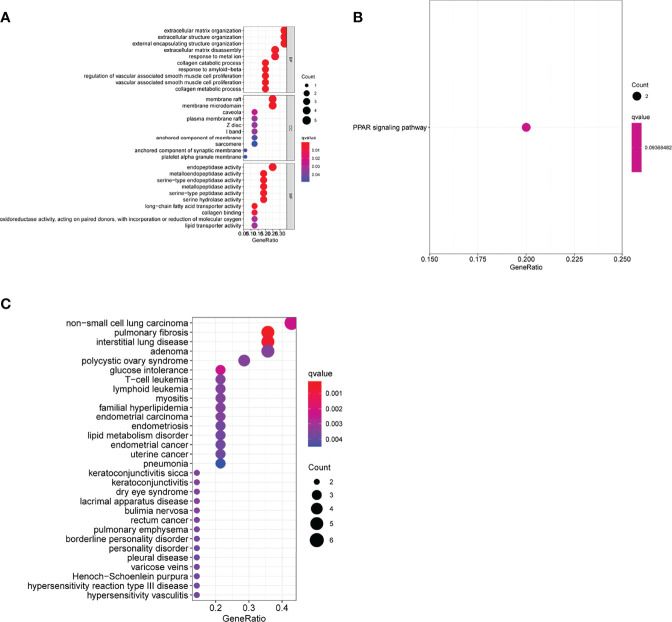
Function enrichment analyses of differentially expressed genes (DEGs). **(A)** Gene Ontology enrichment for DEGs. **(B)** Kyoto Encyclopedia of Genes and Genomes pathways enrichment for DEGs. **(C)** Enrichment study of genes differentially expressed between atherosclerosis and healthy samples using the disease ontology.

We performed a functional correlation analysis to determine the features of the above-mentioned 17 DEGs in life activity. The consequences of the GO analysis showed that the 17 DEGs might be mainly abundant in the extracellular matrix organization, extracellular structure organization, external encapsulating structural organization, membrane raft, membrane microdomain, caveola, endopeptidase activity, serine-type endopeptidase as well as metalloendopeptidase activity (**Figure 2A**). The KEGG assays indicated that the 17 DEGs were mainly enriched in the PPAR signaling pathway (**Figure 2B**). The DO pathway enrichment analyses showed that diseases enriched by DEGs were mainly associated with non-small cell lung carcinoma, pulmonary fibrosis, polycystic ovary syndrome, glucose intolerance, and T-cell leukemia (**Figure 2C**).

### Identification and Validation of Diagnostic Genes

**Figure 3:**
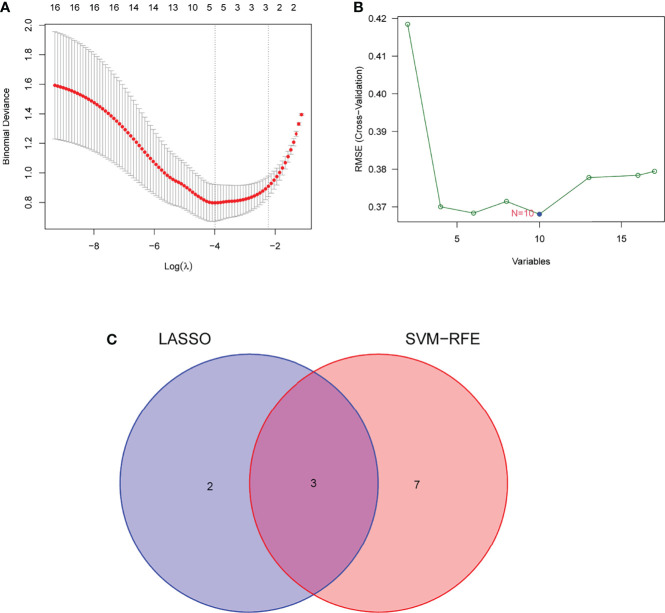
Filtering course in diagnostic candidates in atherosclerosis. **(A)** Tuning character option in the least unconditional shrinkage and option operator model. **(B)** A plot of biomarker option by support vector machine recursive feature elimination (SVM-RFE) algorithm. **(C)** The least unconditional shrinkage, option operator, and SVM-RFE algorithms all share a Venn diagram to provide three diagnostic indicators.

Two distinct algorithms were applied to filter underlying markers. The DEGs were decreased with the LASSO regression algorithm, which confirmed five genes to be diagnostic biomarkers of AS (**Figure 3A**). Apart from the five characters in the DEGs that were decided with the SVM-RFE algorithm (**Figure 3B**), 3 overlapping characters (FHL5, IBSP, and SCRG1) in the 2 algorithms were finally chosen (**Figure 3C**).

### Expression and Diagnostic Value of FHL5, IBSP, and SCRG1 in AS

**Figure 4:**
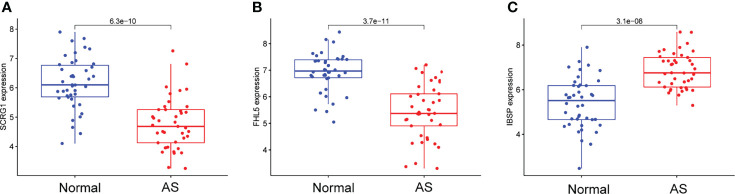
Expression of **(A)** SCRG1, **(B)** FHL5, and **(C)** IBSP, respectively, in atherosclerosis samples and healthy samples from GSE43292 and GSE57691 datasets.

**Figure 5:**
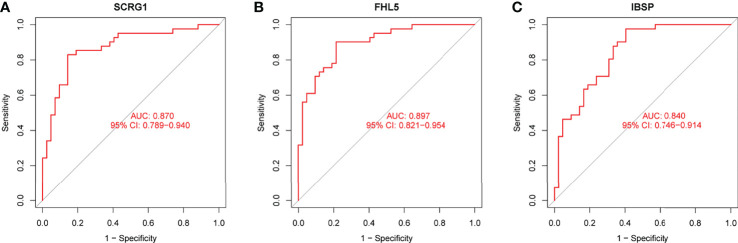
Receiver operating characteristic curve of the diagnostic validity of **(A)** SCRG1, **(B)** FHL5, and **(C)** IBSP, respectively.

We observed that SCRG1 and FHL5 decreased in AS samples compared with those in control samples (**Figures 4A, B****)**, while IBSP expression was distinctly increased in AS samples (**Figure 4C**). In addition, according to the ROC assays, the low SCRG1 conveyed had an AUC result of 0.870 (95% CI: 0.789 to 0.940) in AS (**Figure 5A**), and the low FHL5 expression had an AUC result of 0.897 (95% CI: 0.821 to 0.954) in AS (**Figure 5B**). However, the high IBSP expression had an AUC result of 0.840 (95% CI: 0.746 to 0.914) in AS (**Figure 5C**). Our findings suggested that FHL5, IBSP, and SCRG1 had a high diagnostic ability.

### Correlation of FHL5, IBSP, and SCRG1 With the Proportion of Immune Cell Infiltration

**Figure 6:**
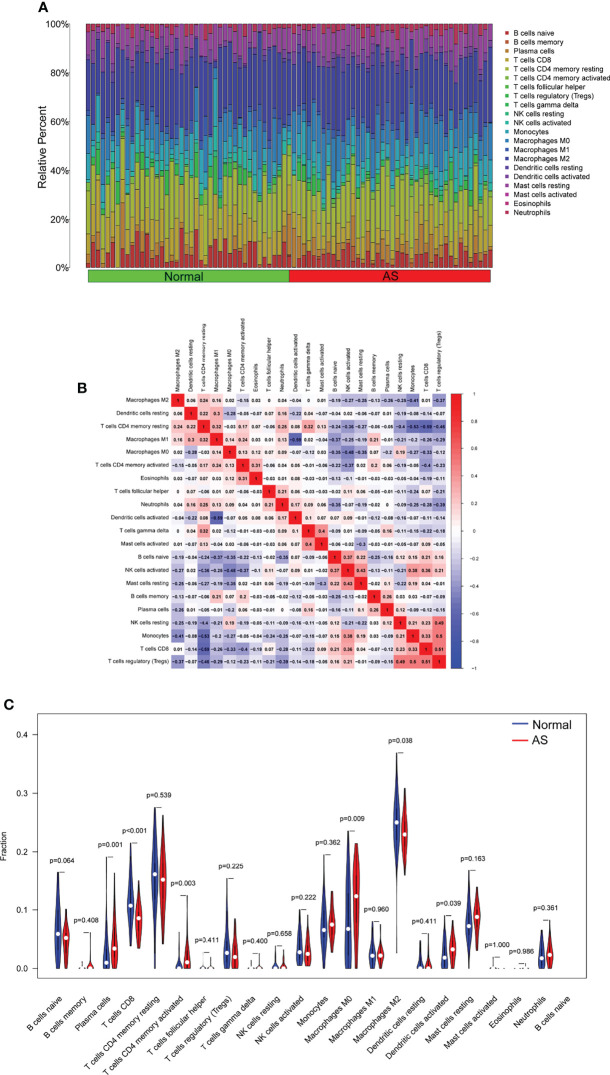
Profile of infiltrating immune cells. **(A)** The ratio of 22 different infiltrating immune cells in atherosclerosis (AS) samples is shown graphically in a bar plot. Column names, sample ID. **(B)** Heat map showing the relationship between 22 different types of infiltrating immune cells. **(C)** Violin plot displaying the variation among 22 types of infiltrating immune cells between AS specimens and healthy samples.

**Figure 7:**
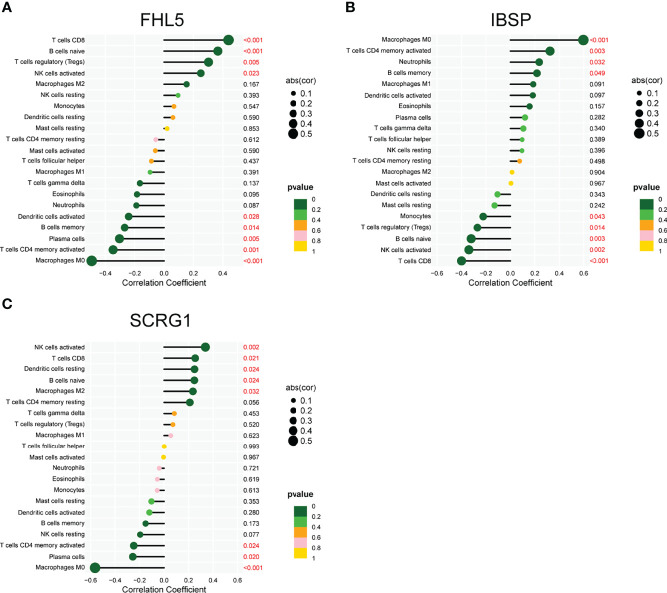
Pertinence between FHL5 **(A)**, IBSP **(B)**, SCRG1 **(C)**, and immune cells of atherosclerosis.

To deeply identify the pertinence in FHL5, IBSP, SCRG1, and the immune microenvironment, the ratio of immune cell infiltration was studied with the CIBERSORT algorithm, and 21 types of immune cell profiling of AS persons were accomplished (**Figures 6A–C**). In addition, it has clear pertinence in FHL5 expression and the ratio in nine types of tumor-infiltrating immune cells (TICs), including CD8 T cells, regulatory T cells (Tregs), naïve B cells, excited NK cells, excited dendritic cells, memory B cells, plasma cells, activated memory CD4 T cells, and macrophage M0 (**Figure 7A** and **Supplementary Figure S1**). It has clear pertinence in IBSP expressions and the ratio in macrophage M0, activated memory CD4 T cells, neutrophils, memory B cells, monocytes, Tregs, naïve B cells, excited NK cells, and CD8 T cells (**Figure 7B** and **Supplementary Figure S2**). In addition, SCRG1 expression was distinctly associated with excited NK cells, CD8 T cells, resting dendritic cells, naïve B cells, macrophage M2, excited memory CD4 T cells, plasma cells, and macrophage M0 (**Figure 7C** and **Supplementary Figure S3**). These results further support the effect of FHL5, IBSP, and SCRG1 on the immune activity of the immune microenvironment.

### Demonstration of the Expression of FHL5, IBSP, and SCRG1 in AS Patients

**Figure 8:**
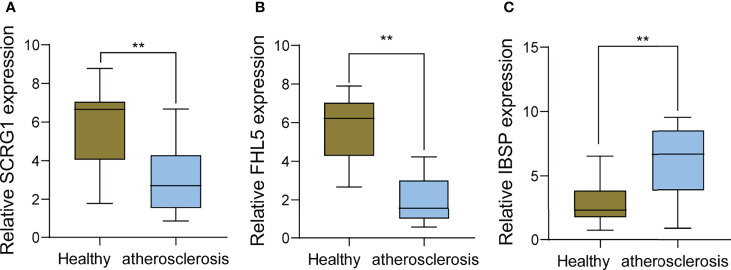
RT-PCR for the expression of **(A)** SCRG1, **(B)** FHL5, and **(C)** IBSP, respectively, in atherosclerosis specimens and healthy samples. **p < 0.01.

To further demonstrate the expression of FHL5, IBSP, and SCRG1 in AS patients, we performed RT-PCR in 10 serum samples from AS patients and 10 healthy samples. The results showed that SCRG1 and FHL5 decreased in AS samples compared with those in healthy samples (**Figures 8A, B****)**, while IBSP expression was distinctly increased in AS samples compared with that in healthy samples (**Figure 8C**).

## Discussion

The number of people with atheroma plaque among 30–70-year-olds worldwide was 57.79 million in 2020, an increase of 59.13% compared with that in 2000 (1, 22). Nevertheless, effective therapy was rare, so it is urgent to identify the genes that result in the development of AS and explore possible efficient treatment targets.

The potential reason for cardiovascular incidents is AS, a chronic inflammatory illness. Accurately diagnosing and treating cardiovascular illnesses necessitated research into the probable processes of AS at the cellular level (23, 24). Because microarrays and high-throughput sequencing have supplied thousands of different gene expression data types, it has become increasingly popular to anticipate prospective treatment targets for atherosclerosis (25, 26). This study retrospectively analyzed data from 42 non-AS arterial and 41 AS arterial samples from two GEO datasets (GSE43292 and GSE57691). We identified 17 abnormally expressed genes between healthy and atherosclerotic tissues. Then, we performed KEGG assays which indicated that the above-mentioned 17 genes might be primarily enriched in the PPAR signaling pathway, which has been reported to be related to the regulation of cardiovascular illnesses (27, 28). The DO assays showed that the above-mentioned 17 genes might be primarily associated with fibrosis, familial hyperlipidemia, lipid metabolism disorder, and several cancers. Our findings suggested that the 17 abnormally expressed genes may be involved in the progression of AS.

SVM-RFE is favorable to small-specimen-size datasets. SVM-RFE also removes spare elements and keeps just the results of the involved variables (29). LASSO regression analysis is always used to screen variables to stop overfitting (30). This study used the above-mentioned two machine learning algorithms to screen possible diagnostic biomarkers for AS, and three genes were identified, including FHL5, IBSP, and SCRG1. Previously, several studies have reported that FHL5, IBSP, and SCRG1 were involved in the progression of several diseases (31–33). However, their effects on AS progression were largely indistinct. In this paper, we first put forward evidence that the expression of FHL5 and SCRG1, respectively, were distinctly decreased in AS samples compared with those in control samples, while IBSP exhibited an increased level in AS samples for the first time. The ROC assays further confirmed their diagnostic value in distinguishing AS from the healthy samples. Our findings highlighted the potential of FHL5, IBSP, and SCRG1 to be used as novel biomarkers for AS patients.

Cells of inborn and adapted immune systems consist of atherosclerotic lesions (34). Infiltrating immune cells have recently been characterized in mouse and human atherosclerosis and revealed activated, cytotoxic, and possibly dysfunctional and exhausted cell phenotypes (35, 36). The chronic accumulation of vascular occlusion plaques in the subendothelial intimal layer of large and medium arteries leads to significant stenosis that restricts blood flow and causes critical tissue hypoxia (37, 38). The autoimmune reply is determinable in human and animal models of AS. While the typical percept is that autoimmunity can be pathogenic *per se*, the present proof indicates that ApoB-specific CD4^+^ T-helper cells have already been determined among subjects without clinical atherosclerosis and with atheroprotective characters mostly. In the paper, it was indicated that FHL5 expression was distinctly related to nine types of TICs, including CD8 T cells, regulatory T cells (Tregs), naïve B cells, activated NK cells, excited dendritic cells, memory B cells, plasma cells, activated memory CD4 T cells, and macrophage M0. IBSP expression was distinctly associated with macrophage M0, activated memory CD4 T cells, neutrophils, memory B cells, monocytes, regulatory T cells (Tregs), naïve B cells, excited NK cells, and CD8 T cells. Moreover, SCRG1 expression was distinctly associated with excited NK cells, CD8 T cells, resting dendritic cells, naïve B cells, macrophage M2, excited memory CD4 T cells, plasma cells, and macrophage M0. Our findings suggested that FHL5, IBSP, and SCRG1 may influence AS progression *via* regulating the activity of the immune system.

Although our findings support that FHL5, IBSP, and SCRG1 have a high clinical application potential, the study faces some limitations. AS samples were limited in this study, and further demonstration by future studies is necessary. Furthermore, particular biological functions in three genes of AS progression are required to be explored experimentally. Moreover, the potential functions of FHL5, IBSP, and SCRG1 on the immune system need to be further studied.

## Conclusions

Our study identified three diagnostic genes, including FHL5, IBSP, and SCRG1. Drugs targeting FHL5, IBSP, and SCRG1 could be potential immunotherapy for AS patients in the future. Nevertheless, other independent cohorts and functional tests for FHL5, IBSP, and SCRG1 are warranted.

## Data Availability Statement

The datasets presented in this study can be found in online repositories. The name of the repository and the accession numbers can be found below:

https://www.ncbi.nlm.nih.gov/, GSE43292
https://www.ncbi.nlm.nih.gov/, GSE57691

## Ethics Statement

The studies involving human participants were reviewed and approved by Suqian First Hospital. The patients/participants provided their written informed consent to participate in this study.

## Author Contributions

YC conceived and designed the study. ZY, XW, and YL provided equal contributions to the research design, data analysis, and article writing. XW revised the manuscript. All authors contributed to the article and approved the submitted version.

## Funding

This study was supported by the National Natural Science Foundation of China (81970387 and 81200213 to YC), Natural Science Foundation of Beijing (7172181 to YC), and Natural Science Foundation of Suqian (K201918 to ZY).

## Conflict of Interest

The authors declare that the research was conducted in the absence of any commercial or financial relationships that could be construed as a potential conflict of interest.

## Publisher’s Note

All claims expressed in this article are solely those of the authors and do not necessarily represent those of their affiliated organizations, or those of the publisher, the editors and the reviewers. Any product that may be evaluated in this article, or claim that may be made by its manufacturer, is not guaranteed or endorsed by the publisher.

## Supplementary Material

Supplementary Figure 1 Nine different types of infiltrating immune cells were linked to FHL5 expression in a scatter plot.

Supplementary Figure 2 Nine different types of infiltrating immune cells were linked to IBS expression in a scatter plot.

Supplementary Figure 3 Eight different types of infiltrating immune cells were linked to SCRG1 expression in a scatter plot.
