# Supplementary figures and images for: The Integrated Analysis Identifies Three Critical Genes as Novel Diagnostic Biomarkers Involved in Immune Infiltration in Atherosclerosis
Source: Front Immunol. 2022 May 18;13:905921. doi: 10.3389/fimmu.2022.905921 (PMC9159807; doi:10.3389/fimmu.2022.905921)

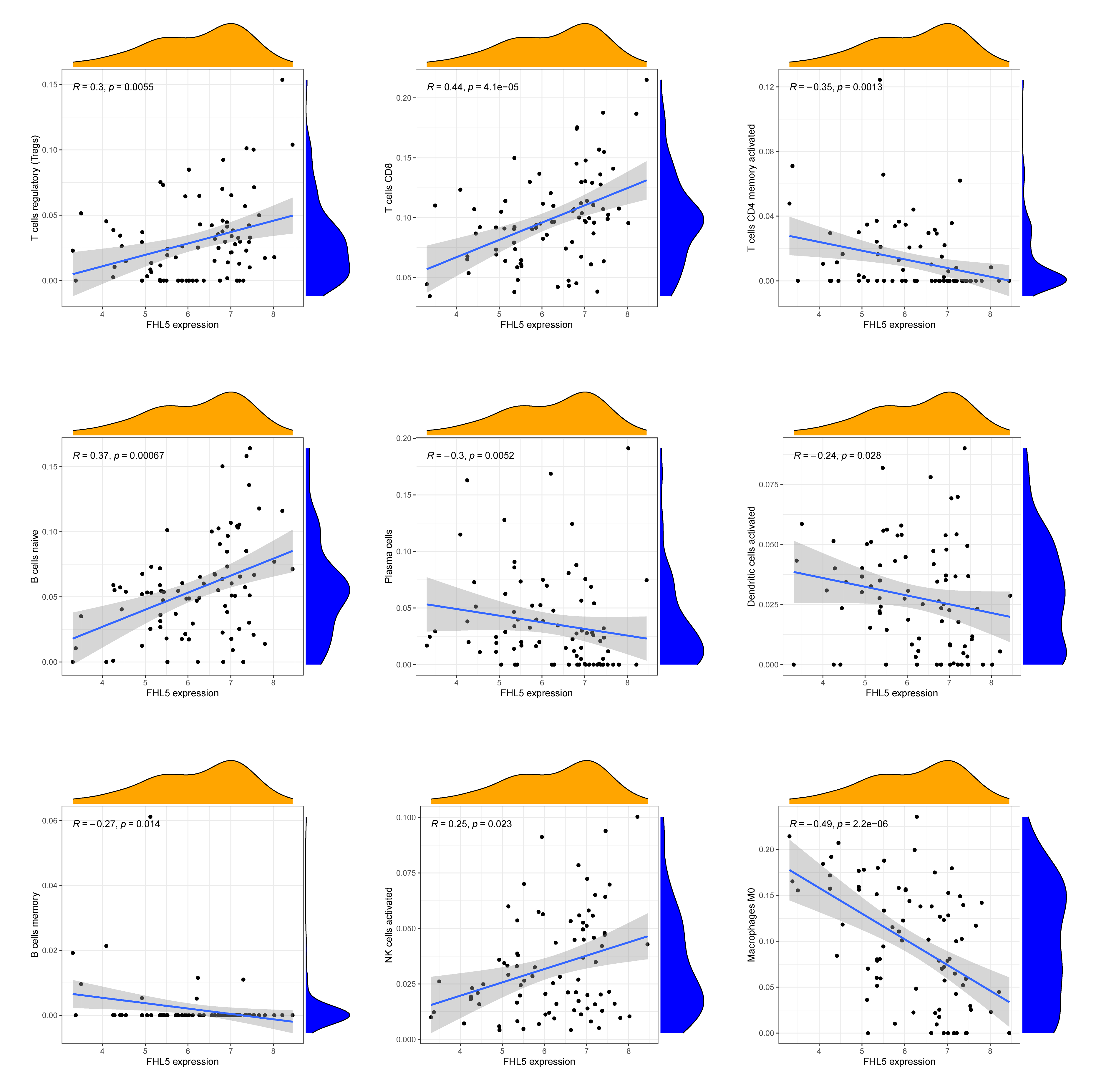

Supplement: Supplementary Figure 1 — Nine different types of infiltrating immune cells were linked to FHL5 expression in a scatter plot. [file Image_1.tif]

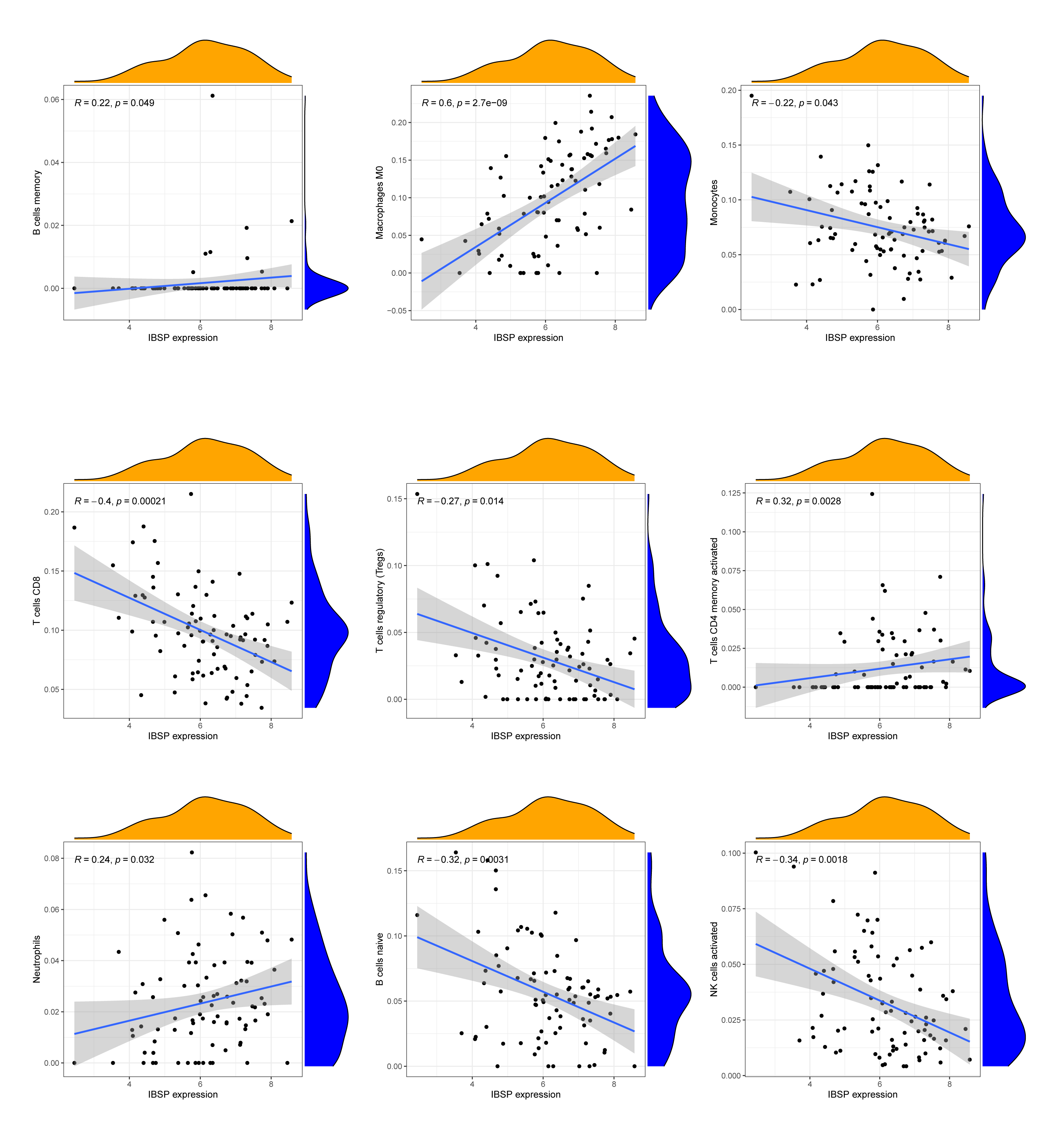

Supplement: Supplementary Figure 2 — Nine different types of infiltrating immune cells were linked to IBS expression in a scatter plot. [file Image_2.tif]

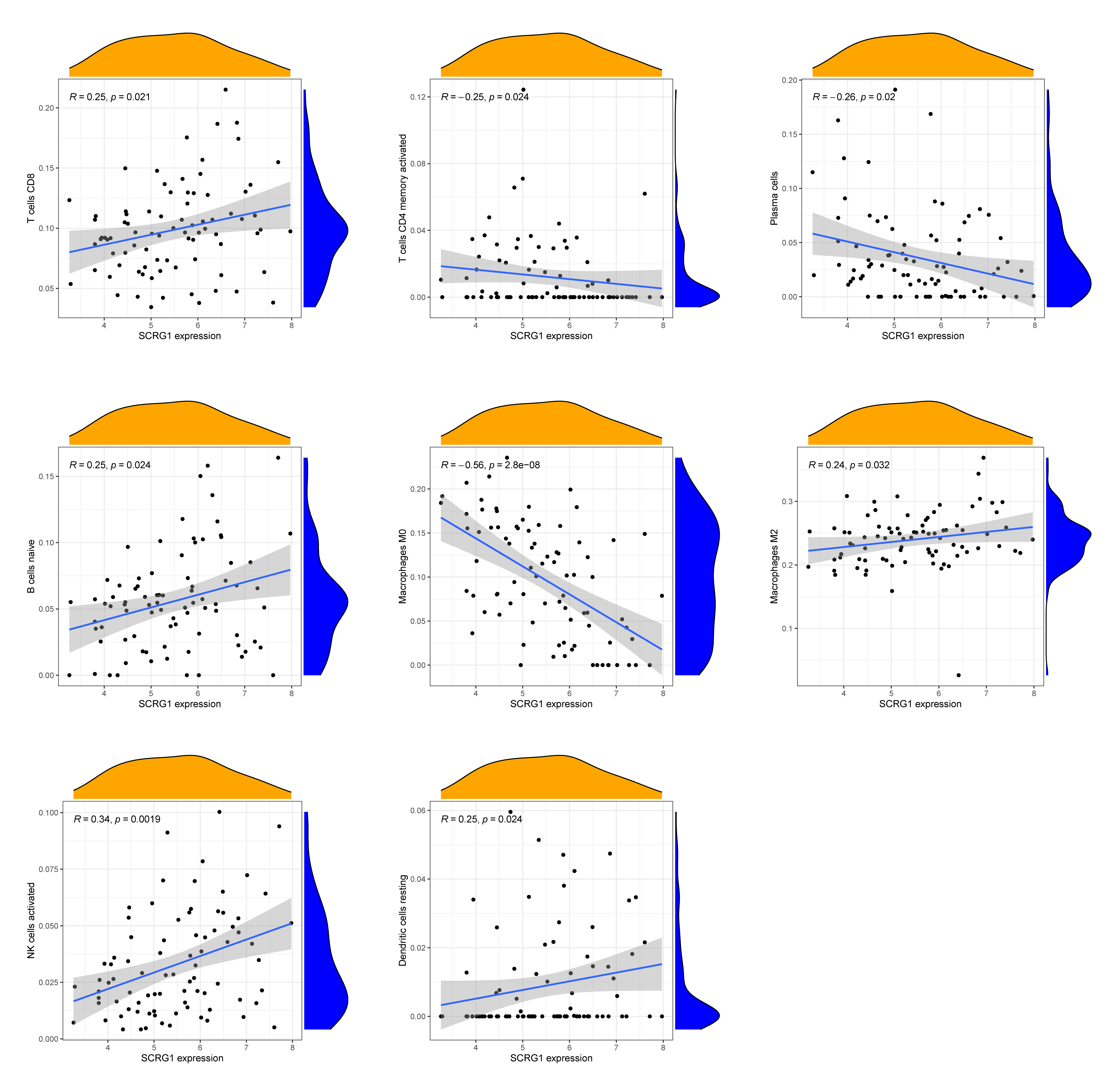

Supplement: Supplementary Figure 3 — Eight different types of infiltrating immune cells were linked to SCRG1 expression in a scatter plot. [file Image_3.tif]
