# Supplementary material for: The Integrated Analysis Identifies Three Critical Genes as Novel Diagnostic Biomarkers Involved in Immune Infiltration in Atherosclerosis
Source: Front Immunol. 2022 May 18;13:905921. doi: 10.3389/fimmu.2022.905921 (PMC9159807; doi:10.3389/fimmu.2022.905921)
Supplement: Supplementary file 4 [file Table_1.docx]

**Table S1** The primer sequences included in this study.

| Name | primer sequences (5’–3’) |
| --- | --- |
| FHL5: forward | CACGGAGTGCTATTCTAACGAG |
| FHL5: reverse | GTTTCATGCCAGTAGTTTCCCTT |
| IBSP: forward | CACTGGAGCCAATGCAGAAGA |
| IBSP: reverse | TGGTGGGGTTGTAGGTTCAAA |
| SCRG1: forward | CCTTGGGCTAACTTTGCTGTT |
| SCRG1: reverse | TGGACATTTGCATCTATCAGCTT |
| GAPDH: forward | GGAGCGAGATCCCTCCAAAAT |
| GAPDH: reverse | GGCTGTTGTCATACTTCTCATGG |
